# **Reproduction in agouti (*Dasyprocta spp***.**): A review of reproductive physiology for developing assisted reproductive techniques**

**DOI:** 10.21451/1984-3143-AR2018-0058

**Published:** 2018-12-05

**Authors:** Érica Camila Gurgel Praxedes, Gislayne Christianne Xavier Peixoto, Andréia Maria da Silva, Alexandre Rodrigues Silva

**Affiliations:** Laboratory on Animal Germplasm Conservation, Universidade Federal Rural do Semi Árido (UFERSA), BR 110, Km 47, Costa and Silva, Mossoró, RN, Brazil.

**Keywords:** biobank, rodentia, wildlife

## Abstract

*Dasyprocta spp.* (agouti) include wild rodents with highlighted ecological and economic importance, and are considered experimental models for endangered hystricognath rodents. Of late, development of techniques to conserve their genetic material as well as the formation of biobanks is increasing. In this context, this review describes the main advances in the knowledge of the reproductive morphophysiological specificities of agouti as well as the development and improvement of assisted reproductive techniques aimed at conservation, multiplication, and exploitation of their reproductive potential under captivity.

## Introduction

**Figure1:**
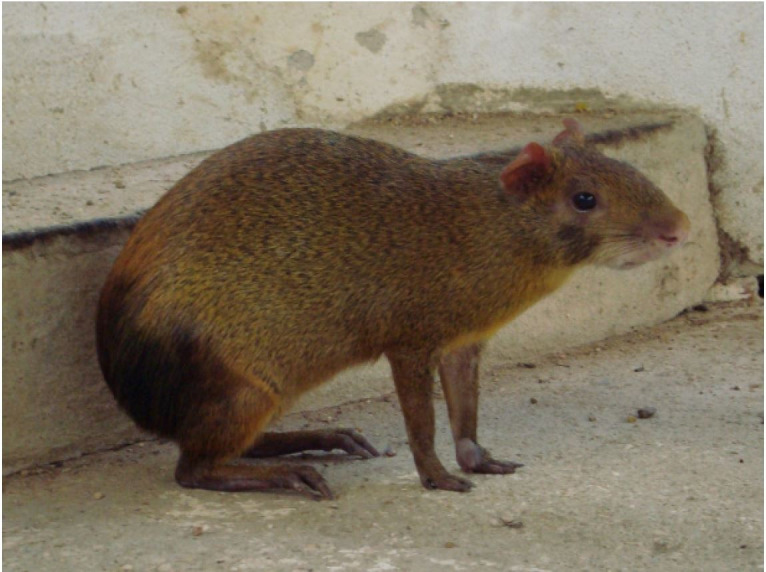
A red-rumped agouti (*Dasyprocta leporina*) specimen.

Rodents account for the largest order of mammals in the world and are found in the most varied types of habitats ( Emmons and Feer, 1997 ). Of these, agouti ( Fig. 1 ), a hystricognath rodent, *Dasyprocta spp.* or *Dasyprocta aguti *, is found throughout the area of Neotropical America and is distributed into 13 different species that constitute the genus *Dasyprocta* ( IUCN, 2018 ).

Agouti is currently suggested as an experimental model for other species of hystricognath rodents that are vulnerable to extinction ( Costa and Martins, 2008 ) because they can easily adapt to captivity and, thus, exhibit prolificity, precocity, and relatively short gestation period ( Hosken and Silveira, 2001 ). These characteristics facilitate studying their physiology and exploring their zootechnical potential ( Pachaly *et al*., 1999 ; Ribeiro *et al*., 2008 ).

Owing to their diverse and substantial distribution, these wild rodents represent an important protein source for human consumption ( Lopes *et al*., 2004 ) as well as for obtaining skin, leather, and bristle ( Silva *et al*., 2010 ). Moreover, they are important seed dispersers and maintain the ecological balance ( Silva *et al*., 2013 ).

Still, with their economic and ecological relevance, agouti present a potential market that justifies the research focused on its conservation ( Souza *et al*., 2003 ) to improve reproduction management of the commercial breed ( Bonaudo *et al*., 2005 ). In this context, this review describes the main advances in research on the reproductive morphophysiological specificities of agouti as well as the development and improvement of assisted reproductive techniques (ART) aimed at conservation, multiplication, and exploitation of their reproductive potential under captivity.

## General characteristics of male and female agouti

The term agouti, also called *Dasyprocta spp.*, refers to the third largest frugivorous rodent ( Henry, 1999 ). The *Dasyprocta* genus comprises approximately 13 species of agouti, viz., *D. azarae*, *D. coibae*, *D. croconota* , *D. fuliginosa*, *D. guamara*, *D. iacki* , *D. kalinowskii*, *D. leporina*, *D. mexicana *, *D. prymnolopha*, *D. punctata*, *D. ruatanica*, and *D. variegata* ( IUCN, 2018 ). The main differences found between these species are related to coat coloration and their conservation status ( IUCN, 2018 ; Table 1 ). However, some species are not easily recognizable owing to taxonomic limitations and absence of any modern taxonomic revision ( Emmons and Feer, 1997 ). Cytogenetic studies are essential because each species is characterized by a typical karyotype, which may differ from others with respect to form, size, and number of chromosomes ( Lima, 2000 ). By cytogenetic analysis of 30 animals of the genus *Dasyprocta* (* D. prymnolopha*, *D. leporina*, and *D. fuliginosa* ), Ramos *et al.* (2003) identified that the individuals presented two cellular lineages, with 2n = 64 and 2n = 65 chromosomes. The karyotypes showed similarity, and chromosomal polymorphism was not detected by Giemsa conventional staining and G banding. All analyzed specimens presented a diploid number of 64 or 65 chromosomes. This variation was observed with a frequency of approximately 70% in cells with 2n = 64 and 30% in cells with 2n = 65. There was no variation in the pattern of nucleolus organizer regions (NORs) in the species studied, which was used to verify chromosomal polymorphism ( Ramos *et al*., 2003 ).

**Table 1:** Main differences among agouti’s species (*Dasyprocta spp*.)

Species	Coats	Conservation status
*Dasyprocta azarae*	Black or white or pale orange	Listed as potentially vulnerable in Argentina
*Dasyprocta coibae*	Cream or black	Vulnerable
*Dasyprocta leporina*	Red-rumped	Estable
*Dasyprocta iacki*	-	Insufficient data
*Dasyprocta guamara*	Dark	Near threatened
*Dasyprocta kalinowskii*	-	Insufficient data
*Dasyprocta croconota*	-	Insufficient data
*Dasyprocta fuliginosa*	Black	Least Concern
*Dasyprocta mexicana*	Mexican Black	Critically Endangered
*Dasyprocta prymnolopha*	Black-rumped	Least Concern
*Dasyprocta punctata*	Uniform reddish brown	Least Concern
*Dasyprocta ruatanica*	Black or Cream	Endangered
*Dasyprocta variegata*	Brown	Insufficient data

Source: IUCN, 2018.

Agouti are distributed over a wide area of Neotropical America and are found in a great diversity of habitats from the south of Mexico, through Central America, to Argentina, Uruguay, and Paraguay ( Deutsch and Puglia, 1988 ). They exhibit an extraordinary variety of ecological adaptations, supporting existence in the coldest and more torrid climates. They can thrive in regions with the highest floristic cover at high altitudes ( Emmons and Feer, 1997 ).

Agouti are medium-sized animals, weighing between 2 and 5 kg, with an average height of 23 cm and length of 50 cm, measured from the muzzle to the base of the tail, with the tail being generally bristle. The head and thorax show fur colors ranging from gray to bright orange covered by long, harsh, and strong pelage, with predominating brown and light or dark yellowish or golden colors. The top of the head, neck, and middle of the back between the shoulders are sometimes dark with long bristles; the ears are small and bare, similar to the tail that is almost vestigial ( Smythe, 1987 ; Emmons and Feer, 1997 ). In addition, they possess four long and curved incisor teeth, whose macroscopic characteristics reveal structure similar to that of other mammals ( Oliveira *et al*., 2012 ). Between males and females there is no sexual dimorphism; therefore, to differentiate them, it is necessary to perform sexing through palpation, where the difference between the male’s prominent penis and the female’s two vaginal folds, forming the vulva, is easily observed ( Guimarães *et al*., 2009 ).

These animals have adapted to terrestrial life by a reduction in functional fingers and a vestigial thumb, with four digits on the forelimbs and three on the hindlimbs. Furthermore, the claws of the forelimbs are slightly arched, indicating the ability to excavate, although they are not true diggers. The hindlimbs are much larger than forelimbs, enabling them to jump ( Oliveira *et al*., 2012 ).

These animals have diurnal and crepuscular habits, and when threatened, they may present nocturnal habits. They are naturally herbivorous feeding on leaves, roots, flowers, fungi, seeds, and, particularly fruits found in the soil ( Santos *et al*., 2005 ). In case of fruit shortage, agouti spend less time resting and more time foraging, looking for seeds that have been buried previously ( Smythe, 1987 ). Such behavior of digging, hiding, and seeking, also observed in *Myoprocta spp* ., is essential for the dispersion of plant species ( Forget and Milleron, 1991 ). Thus, agouti act on aggregate distribution of tree seeds that is secondary to the action of long-distance dispersal animals, such as tapirs (*Tapirus terrestris*) (Lange and Schmidt, 2007). These species are also important links in the food chain because they are prey to birds, snakes, and wild carnivores, thereby maintaining the environmental balance ( Hosken and Silveira, 2001 ). In addition, agouti are economically important because they enable the commercialization of skin, leather, and bristle ( Silva *et al*., 2010 ). Yet, to their economic and ecological importance, because of their stable world population, they can be used as experimental models for endangered agouti, such as *D. ruatanica *, *D. coibae*, and *D. mexicana* ( IUCN, 2018 ).

Because they are considered as important protein sources, agouti have been used for human consumption. Their meat is white, rich in nutrients, soft, tasty, and an excellent source of calcium and phosphorus, with low caloric value (each 100 g of meat has approximately 120 Kcal) (Barbosa, 2010). The commercialization of agouti meat provides low market value, requiring little manpower. The system of fattening and development of matrices are two activities that can be practiced at the same time, potentiating producer profit. According to some researchers, the meat of these animals is as appreciated as paca meat, the most sought-after in the market among the wild species (Barbosa, 2010). For example, in Trinidad and Tobago, and Central America, agouti meat is considered a spice; therefore, this animal has been intensively hunted ( Roopchand, 2002 ).

Breeding agouti under captivity is an alternative for its preservation in the natural habitat and allows the exploration of their zootechnical potential, thereby allowing studies on their physiological characteristics ( Pachaly *et al*., 1999 ; Lopes *et al*., 2004 ). However, studies with the appropriate methods of productive and reproductive management of wild animals are limited, which could reflect in higher productivity, enabling the preservation of some species by reduction in predatory hunting ( Lopes *et al*., 2004 ).

Hence, it is necessary to apply captive breeding programs and ART as tools to ensure the increase in their reproductive potential and conservation of their genetic material ( Comizzoli *et al*., 2001 ), allowing the increase in their zootechnical potential and genetic variability; thus, enabling the study of their physiological characteristics.

### Reproductive morphophysiological characteristics of male agouti

In agouti, the internal reproductive system is composed of accessory glands (vesicular glands, prostate, and bulbourethral glands), testis, and epididymis (delimited by adipose tissue, i.e., caput epididymal) that are paired and fully coated by cremaster muscle, allowing a greater range of movement and internalization of the testicle in the abdomen ( Paula and Walker, 2012 ). In addition, the epididymis of *Dasyprocta spp*. is composed of principal, basal, halogen, apical, and clean cells with pseudostratified, columnar, and stereociliated epithelium ( Arroyo *et al*., 2014 ).

According to Mollineau *et al.* (2006) in *D. leporina*, the testicular length, diameter, and weight are 3.67 ± 0.12 cm, 1.67 ± 0.04 cm, and 5.03 ± 0.52 g, respectively. In addition, the ductus deferens has a length of 10.98 ± 0.40 cm and mean diameter of 0.14 ± 0.01 cm. The absence of a scrotum, a characteristic observed in agouti, has been reported by Menezes *et al.* (2003) .

**Figure 2:**
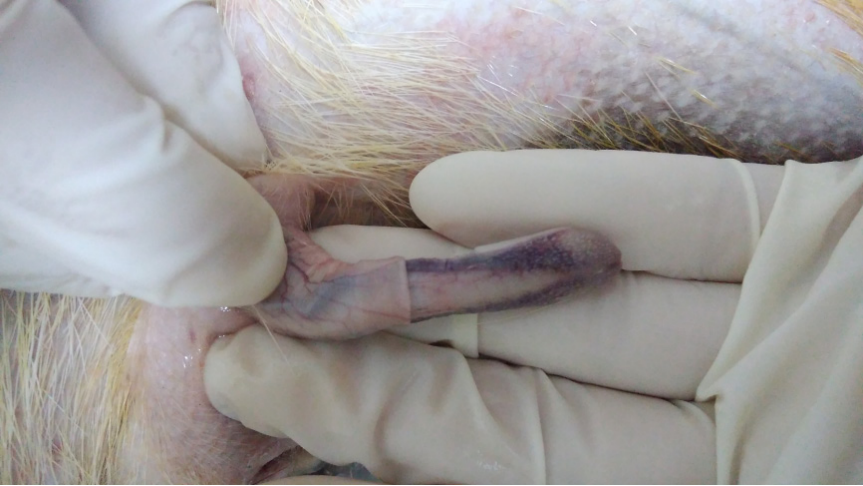
Agouti’s (*Dasyprocta leporina*) penis with prepuce partly removed.

The penis ( Fig. 2 ) is doubled caudally with a U-bend lying down, with a mean length of 9.90 ± 0.43 cm, and contains paired ventral keratinous spicules. It is observed that the glans penis presents a rounded dilation, called urethral torus or penile flower, at the time of erection ( Carvalho *et al*., 2008 ; Mollineau *et al*., 2012 ). Four steps are documented in the erection process: stage 1, protrusion of the penis from the preputial orifice; stage 2, opening of the lateral cartilages of the penis; stage 3, flowering of the head of the glans penile flower, eversion of the intromitting bag, and protrusion of the keratinous spicules; and stage 4, protrusion of the keratinous spurs and ejaculation ( Mollineau *et al*., 2012 ).

Regarding reproductive development in males, Costa *et al.* (2010) observed that the spermatogenic cycle lasts for 9.5 ± 0.03 days, and the total duration of spermatogenesis is 42.8 ± 0.16 days. According to Assis Neto *et al* . (2003a), the period from birth to five months comprises the pre-pubertal period, in which the presence of gonocytes is observed with the absence of tubular lumen in the testicular cords as well as the presence of spermatogonia, spermatocytes, spermatids, Sertoli and Leydig cells ( Arroyo *et al*., 2017 ); from six to eight months of age is the transition phase (pre-puberty to puberty), in which 40% to 90% of the testicular cords are in the process of tubular lumination, coinciding with the appearance of the first primary spermatocytes and rounded spermatids. Puberty in male agouti is established at around the age of nine months, where it is possible to observe the presence of all cell types and spermatozoa in the testicular lumen after eight stages of the cycle of the seminiferous epithelium (Assis Neto *et al*., 2003b). According to Arroyo *et al*., (2017) , the main changes in the testis of agouti (*Dasyprocta spp.)* occur between the prepubescent and prepubertal periods, when the germinal epithelial organization occurs and the Sertoli cells undergo morphological and functional changes to form the spermatozoa.

**Figure 3:**
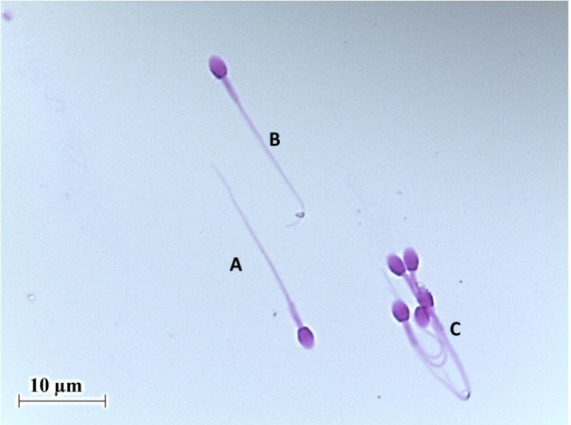
Morphology of agouti’s (*Dasyprocta leporina*) epididymal sperm stained with Bengal rose. (A) Normal spermatozoa; (B) abnormal spermatozoa with tail folded; (C) abnormal spermatozoa with high tail folded.

Morphological analysis of agouti *(Dasyprocta spp.)* sperm demonstrated that these cells present an oval-shaped head, like a shovel with a rounded head apex, and a flat base ( Fig. 3 ). Sperm head is tapered, without prominence of the acrosome or evidence of the perforatorium ( Arroyo *et al*., 2017 ). Likewise, it has been reported that the base of the head is symmetric and the tail is extended and sharpened ( Mollineau *et al*., 2006 ; Ferraz *et al*., 2009 ). Regarding the morphometry, the sperm presented distinct results among species, 48.0 ± 0.3 μm and 40.12 ± 2.4 μm for the species *D. leporina* and *D. prymnolopha*, respectively ( Mollineau *et al*., 2008 ; Ferraz *et al*., 2011 ).

In this context, in wild animals, it is necessary to determine the anatomy and physiology of the species before implementing ART, to adapt to the characteristics of each species. Further, it is often necessary to perform tests of interaction between different factors that may affect the success of the technique.

### ART for male agouti

Application of ART as an initial step for developing methods for sperm recovery is required. Of late, methods for obtaining spermatozoa by retrograde epididymal washing ( Ferraz *et al*., 2011 ; Silva *et al*., 2011 , 2012 ; Castelo *et al*., 2015 ) or electroejaculation ( Mollineau *et al*., 2008 ; Martinez *et al*., 2013 ; Castelo *et al*., 2016 ) have been described for agouti.

Obtaining epididymal spermatozoa offers the possibility of acquiring and using the genetic material immediately after the animal’s death ( Goovaerts *et al*., 2006 ) because the genetic material is morphologically viable with a suitable degree of maturity and fertilizing capacity ( Monteiro *et al*., 2011 ). There are two basic methods for obtaining spermatozoa from the epididymis, viz., retrograde washing and flotation. The most used method is retrograde washing ( Martinez-Pastor *et al*., 2006 ). This method consists of injecting an aqueous medium followed by tail cutting, in the direction from the ductus deferens to the tail epididymis, for the recovery of sperm ( Comizzoli *et al*., 2001 ). In agouti, Ferraz *et al.* (2011) were the first to demonstrate the possibility of obtaining spermatozoa by means of retrograde washing in animals previously submitted to orchiectomy, and they obtained a concentration of 748 ± 418.66 spermatozoa/mL. Subsequently, the technique was applied in the same species of agouti by Silva *et al.* (2011 , 2012 ), in which a volume of 300 ± 2 μL was recovered with a concentration of 1.4 ± 0.3 × 10^9^ spermatozoa/mL. Using the same method in *D. leporina *, Castelo *et al.* (2015) were able to recover a total volume of 1.65 ± 0.22 mL with a concentration of 1.04 ± 0.2 × 10^6^ spermatozoa/mL.

Electroejaculation is another efficient way of obtaining sperm, primarily because the death of the animal is not necessary. This method of semen collection is based on the induction of ejaculatory reflex through the application of electrical stimuli on the nerve plexus located in the pelvic floor of the animal. A lubricated transrectal probe connected to an electrical stimulator is introduced in the rectum of the animal under anesthesia or without anesthesia to achieve stimuli ( Silva *et al*., 2004 ). Mollineau *et al.* (2008) were the first to describe the use of electroejaculation in *D. leporina* using ketamine as an anesthetic; they applied a charge of 6 V for 5 s followed by increasing the charge by 1 V until 12 V with 5 s intervals of rest. Thus, the authors obtained an efficiency of 30% of ejaculates containing spermatozoa. In a subsequent study using the same electroejaculation protocol, but starting at 2 V, an efficiency of 40% of ejaculates containing spermatozoa was obtained ( Mollineau *et al*., 2010a ). Additionally, Castelo *et al.* (2016) demonstrated the interaction between different types of electrical stimuli, as sine or quadratic waves, and electroejaculation using ring or strip electrode apparatus with better results than those reported by Mollineau *et al.* (2008 , 2010). The authors obtained 70% of ejaculates, with 57% of ejaculates containing spermatozoa, when using the protocol with the ring electrode associated with sinusoidal stimuli ( Castelo *et al*., 2016 ). However, Martinez *et al*. (2013) obtained better results using four brown agouti (*D. azarae*) and obtained 100% success in semen collection. For this, the authors used an association of azaperone and meperidine as preanesthetic medication. Afterwards, the animals were anesthetized with the combination of ketamine and xylazine, followed by lumbosaccharide application of lidocaine. The protocol of stimulation comprised four sets of 20 electrostimulations for 3 s each with 2, 4, 6, and 8 V, with a 2-min interval between each series. The results of evaluation of sperm characteristics obtained from such experiments are shown in Table 2 .

**Table 2:** Values (Mean ± SEM) for the agoutis’ (*D. Leporina* ) sperm parameters obtained by electroejaculation and retrograde epididymal washing

Sperm parameters	Eletroejaculation	Retrograde epididymal washing
Volume (mL)	0.6 ± 0.1	1.65 ± 0.22
Sperm concentration (× 10^9^ sperm/mL)	307.5 ± 69.6	822.5 ± 85.0
Sperm motility (%)	93.7 ± 4.7	96.2 ± 2.4
Vigor (0-5)	5.0 ± 0.0	5.0 ± 0.0
Membrane integrity (%)	74.0 ± 4.0	90.5 ± 2.1
Osmotic response (%) Sperm morphology (%)	66.2 ± 4.0 77.2 ± 4.1	79.7 ± 2.6 80.7 ± 8.1

Source: Castelo (2015) .

With respect to the development of preservative protocols, Silva *et al.* (2011) evaluated the performance of Tris and powdered coconut water (ACP-109c) diluents on the cryopreservation of epididymal sperm derived from *D. leporina*, wherein the samples were centrifuged, and extended in the same diluents in addition to egg yolk (20%) and glycerol (6%). After sperm cryopreservation and thawing, they observed that 26.5 ± 2.6% were motile sperm with 2.6 ± 0.2 vigor in the ACP-109c group, which was significantly better than 9.7 ± 2.6% motile sperm with 1.2 ± 0.3 vigor found in the Tris group. They verified that ACP-109c would be the most suitable diluent for processing and cryopreservation of these cells. Subsequently, the same group, studying the interactions between straw size (0.25 or 0.50 mL) and thawing rates (37°C for 60 s or 70°C for 8 s) for epididymal sperm, demonstrated that epididymal sperm of agouti could be efficiently cryopreserved in both 0.25 mL or 0.50 mL straws and thawing should be conducted at 37°C for 60 s. The use of 0.25 mL and 0.5 mL straws thawed at 37°C for 60 s provided a value of 26.6% and 18.4% for sperm motility, respectively ( Silva *et al*., 2012 ).

Furthermore, for cryoprotectants (CPAs), Castelo *et al.* (2015) , using glycerol, dimethylsufoxide (DMSO), and dimethylformamide (DMF) at 3% and 6% concentrations, demonstrated that 6% glycerol was the most appropriate for cryopreservation of spermatozoa of *D. leporina* compared to that by other CPAs, in which it was possible to recover spermatozoa with a mean motility of 39.5 ± 4.6% after thawing.

Through electroejaculation, Mollineau *et al*. (2010a) diluted the ejaculates of *D. leporina* in ultra-high-temperature (UHT) milk, unpasteurized coconut water, or pasteurized coconut water under refrigeration at 5°C. After 24 h of storage, best results were achieved in the samples diluted in UHT milk, with sperm motility values of 59.5 ± 7.75%. For cryopreservation, however, only 12.5% of sperms presenting progressive motility were obtained after thawing at 30°C for 20 s using the same milk diluent ( Mollineau *et al*., 2010b ). Recently, Castelo *et al.* (2016) cryopreserved samples derived via electroejaculation conducted on *D. leporina.* They demonstrated that the use of an extender containing ACP-109c with 20% egg yolk and 6% glycerol was able to yield 31.2% motile sperms after thawing.

Recovery of epididymal sperm appears as the most viable alternative for male gamete retrieval in this species to use viable sperms for the development of cryopreservation procedures. However, in agouti, obtaining sperm by electroejaculation is still a challenge, and based on these findings, we can infer that the main obstacle for the improvement of ARTs in male agouti is the low efficiency of electroejaculation protocols. Standardizing these protocols requires studying factors that may affect the success of cryopreservation that are appropriate to the inherent characteristics of each species. In addition to the type of CPAs, there is need for consideration of important factors such as their concentration and effects on sperm fertilizing ability.

Despite the advances already achieved ( Tab. 3 ), the need for further studies is highlighted, with the objective of improving the protocols of electroejaculation with respect to time, interval between series, and anesthetic planes as demonstrated for other domestic and wild species. In the cryopreservation protocols, incorporation of new additives as detergents based on sodium dodecyl sulfate (SDS) and new CPAs, such as *Aloe vera* extract, may improve the quality and longevity of the sperm cell. To our knowledge, cryopreservation of testicular tissue has not been reported, which would represent a method for the conservation of the biodiversity of this species with future application in *in vitro* culture and optimization of diverse reproductive biotechniques.

**Table 3:** State of the art of assisted reproductive techniques (ARTs) applied for agoutis’ (*Dasyprocta spp.)* males.

Species	ART	Source
*Dasyprocta azarae*	Eletroejaculation	Martinez *et al*., (2013)
*Dasyprocta leporina*	Retrograde epididymal washing	Silva *et al*., (2011) Castelo *et al*., (2015)
	Eletroejaculation	Mollineau *et al*., (2008 ; 2010a )
		Castelo *et al*., (2016)
	Refrigeration of sperm	Mollineau *et al*., (2010a ; 2010b )
		Silva *et al*., (2011 ; 2012 )
	Crypreservation of epididymal spermatozoa	Castelo *et al*., (2015)

### Reproductive morphophysiological aspects of female agouti

**Figure 4:**
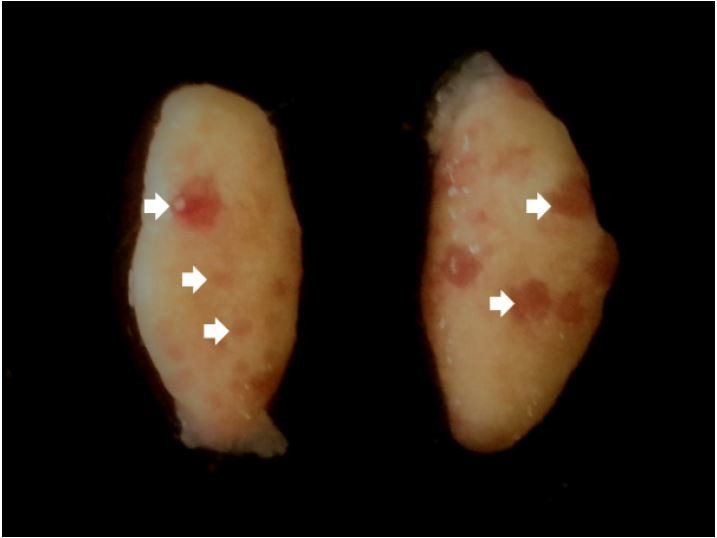
Ovary of the species *Dasyprocyta leporina*, with follicles at various stages of development (white arrows).

The ovaries ( Fig. 4 ) of the agouti are located in the sublumbar region, caudally to the kidney, in the abdominal cavity presenting an ellipsoid or oval shape, flattened dorsoventrally ( Almeida *et al*., 2003 ). According to the morphometric data, the right ovary weighs an average of 0.082 g, with a length of 0.83 cm, width of 0.49 cm, and thickness of 0.24 cm; whereas, the left ovary weighs 0.058 g, with a length of 0.74 cm, width of 0.45 cm, and thickness of 0.23 cm ( Almeida *et al*., 2003 ). Histologically, the ovaries are covered by a simple cubic epithelium on a layer of connective tissue rich in fibers, and a high volume of accessory corpora lutea are described in this species (Weir, 1974). The ovaries are light yellow in color, with a smooth outer surface and small translucent areas, suggestive of the presence of follicles in different categories ( Santos *et al*., 2018 ).

**Figure 5:**
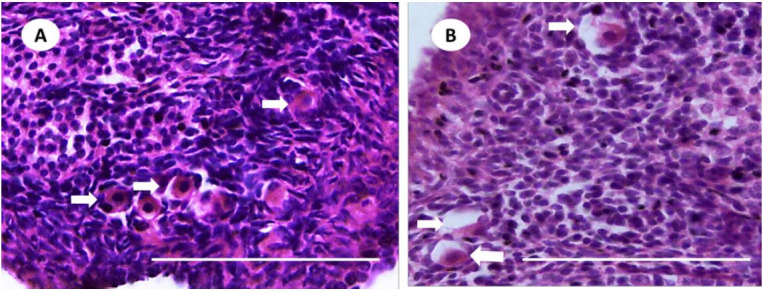
Photomicrographs of agouti (*Dasyprocta leporina*) ovarian sections. (A) Aggregates of primordial follicles displaying an oocyte surrounded by one layer of flattened cells (white arrows). (B) Degenerated follicles displaying oocyte cytoplasm retraction and disorganization of granulosa cells (white arrows).

The follicle, a morphofunctional unit of the ovary, presents dimensions varying between the follicular classes ( Fig. 5 ). The primordial follicle is 18.62 ± 3.40 μm in diameter, with oocyte of 12.28 ± 2.37 μm and nucleus of 6.10 ± 0.93 μm; the primary follicle is 23.75 ± 5.70 μm in length, oocyte of 14.22 ± 3.00 μm, and nucleus of 6.70 ± 1.24 μm; and the secondary follicle is 88.55 ± 17.61 μm in length, oocyte of 52.85 ± 17.56 μm, and nucleus of 22.33 ± 17.61 μm ( Santos *et al*., 2018 ). In addition, the follicular population in *D. leporina* is estimated at 4419.8 ± 532.26 and 5397.52 ± 574.91 follicles in the right and left ovary, respectively. A high number of polyovular follicles, representing 7.51% of the follicles, are observed.

In general, for all females agouti, puberty is established at around the age of nine months; however, further information on the establishment of this event in different species of *Dasyprocta* is limited. The estrous cycle lasts for a mean period of 34.2 ± 2.1 days, with the existence of short cycles of only 12 days ( Weir, 1971 ). However, in *D. prymnolopha*, Guimarães *et al.* (1997) conducted a study through vaginal cytology analyses and verified a mean duration of 30.69 ± 4.65 days for the estrous cycle, with variations between 19 and 40 days. Subsequently, they confirmed their results through hormonal measurements, performed twice a week, and determined the mean duration of the estrous cycle as 32.05 ± 4.17 days. There were no statistical differences in 17β-estradiol levels between the cycle phases. However, two peak periods of 17β-estradiol were observed, the first being in the metestrus (75.88 pg/mL) and the second during the proestrus (77.26 ± 20.71 pg/mL). In the estrus, the initial progesterone concentration is low (2.83 ± 2.34 ng/mL), but an increase in the progesterone level is observed at 24 h (9.02 ± 2.34 ng/mL) ( Guimarães *et al*., 2011 ). For *D. leporina*, the estrous cycle was characterized as being polyestrous continuous with an average duration of 28 days, ranging from 24 to 31 days. Ultrasound analysis revealed no differences in ovarian morphology during the different phases of the estrous cycle. Follicles during the estrogenic phases (proestrus and estrus) were identified with an average diameter of 1 ± 0.5 mm. In only 12.5% of luteal phases, corpora lutea, measuring 1.4 ± 0.9 mm, were identified ( Campos *et al*., 2015 ). The females of these species have a vaginal occlusion membrane, the perineum or operculum, the presence of which enables identification of the estrous phase, as observed in *D. prymnolopha* (Weir *et al*., 1974), and which opens only during the estrous cycle and parturition.

Overall, for all the agouti species, there are few studies on the reproductive morphology and physiology. However, extensive knowledge on the female reproduction is important for the application of auxiliary reproductive biotechniques for its conservation and management.

#### Gestation physiology and monitoring

Kleiman et al. (1979) observed that for agouti, an average gestation period was 112 days, which varied among the 13 existing species. The occurrence of postpartum estrus has been reported in agouti and occurs between 18 and 20 days ( Weir, 1971 ). The placenta is lobulate, monohemochorial, and is connected to the uterus through the mesoplacenta ( Rodrigues et al., 2003 ).

Sousa *et al.* (2012) performed multifrequency sonography (5-7.5 MHz) using a microconvex transducer to observe the characteristics related to the pregnancy age of the agouti *D. prymnolopha *. The first uterine morphological changes were observed on day 9 as an anechoic spherical structure, with slightly hyperechoic margins, and gestational sac was observed at only around 76 days after mating. In a recent study with the same species, B-mode ultrasonography associated with Doppler allowed the evaluation of the vascular network and determination of the reference values for blood flow necessary to maintain fetal viability at different gestational ages ( Sousa *et al*., 2017 ). In addition, morphogenetic analysis of the fetuses of *D. leporina* from 30 to 100 days revealed stages of embryonic and fetal development ( Oliveira *et al*., 2017 ), demonstrating the progress of species-oriented studies.

### ART applied to female agouti

Understanding the estrous cycle of a species is essential for the development of ART ( Durrant, 2009 ). High variability among wild species, duration of the estrous period, and difficulty in determining the exact time of ovulation led to the development of useful methods for monitoring the estrous cycle (Pimentel *et al*., 2014).

**Figure 6:**
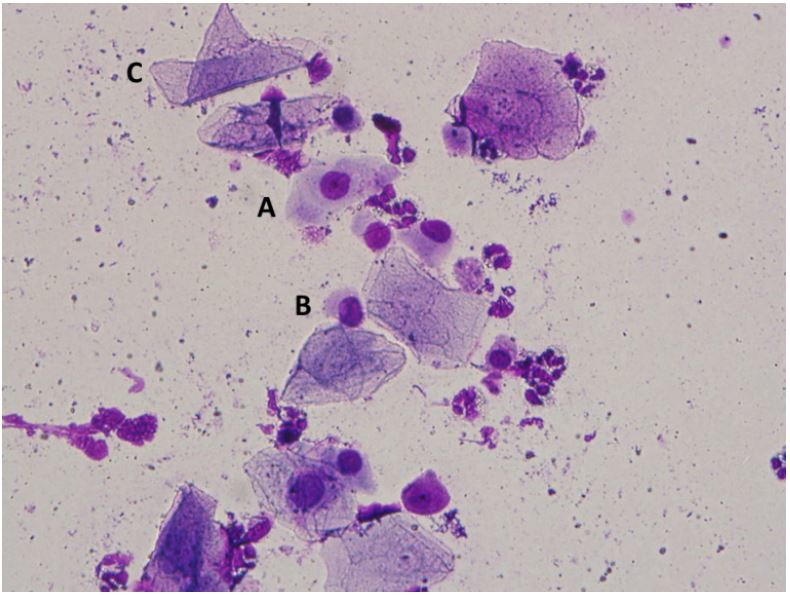
Exfoliative vaginal cytology of agouti (*Dasyprocta leporina*). (A) intermediate cell; (B) parabasal cells; (C) surface cell.

In a study conducted on animals bred in the Caatinga biome, it was possible to characterize the estrous cycle of *D. leporina* by means of exfoliative vaginal cytology ( Fig. 6 ) and ultrasonography. Vaginal cytology revealed the predominance of superficial cells in the stages of proestrus and estrus, followed by intermediate cells in the metaestrus, thereby allowing the distinction of the follicular and luteal phases ( Campos *et al*., 2015 ). Similar cytological findings have also been reported for *D. prymnolopha* ( Guimarães *et al*., 1997 ). Additionally, the external genitalia of *D. leporina* presented changes related to the typical signs of the estrous phase, such as the opening of vulvar lips with the presence of mucous secretion. Such changes coincided with the presence of an ovarian follicle (with an average diameter of 1 ± 0.5 mm, varying from 0.4 to 1.6 mm) as observed by ultrasonography, with the predominance of superficial cells ( Campos *et al*., 2015 ). Furthermore, ultrasonography revealed that there were no differences in ovarian morphology during the different phases of the estrous cycle, but it was possible to observe follicles in the follicular phases and corpora lutea, measuring 1.4 ± 0.9 mm, in the luteal phases ( Campos *et al*., 2015 ). The results of ovarian morphometry detected by ultrasonography in the different phases of the estrous cycle in *D. leporina* were similar to those observed in black agouti (*D. fuliginosa*) ( Mayor *et al*., 2011 ).

The cycles of female *D. leporina* were confirmed by blood hormonal measurements. Females were monitored throughout their estrous cycle, and estrogen (E_2_) and progesterone (P_4_) concentrations were determined at all stages by enzyme linked immunosorbent assay (ELISA). E_2_ levels were 1212-3500 pg/mL, with the peak value coinciding with the observed estrus. However, two additional peak values for E_2_ , one in the metaestrus and one in proestrus were also recorded. The concentrations of P_ 4_ reached a maximum value of 4.23 ng/mL, and the increase in P_4_ occurred immediately after the second peak of E_2_ in metaestrus, with the highest concentrations of P_4_ recorded in diestrus ( Singh *et al*., 2016 ). However, in agouti, there are still few reports on monitoring of the estrous cycle through hormonal dosages.

The induction of estrus was reported for *D. leporina*, in which the hormonal protocols consisting of peritoneal administration of cloprostenol (5 μg) alone or in combination with a gonadotropin-releasing hormone (GnRH) analog (30 μg) were compared. Using only cloprostenol, estrus induction was verified in only 40% of females that manifested estrus signs and presented estrogen peak at 3 and 6 days after drug administration. However, combination therapy with both the hormones yielded estrus induction in 60% of animals, but 40% of the animals manifested estrus at day 4 and 20% at day 10 day after drug administration ( Peixoto *et al*., 2018 ). Although these protocols promoted estrus induction, the authors do not suggest this method as an effective means to achieve synchronization of estrus induction in *D. leporina *.

Parallel to these studies, cryopreservation of ovarian tissue has been developed with the aim of creating biobanks using the female germplasm. For *D. leporina*, preservation of up to 64% preantral follicles (PFs) was achieved using a conventional freezing method with different CPAs, such as DMSO, ethylene glycol (EG), and propanediol (PROH) at 1.5 M. However, transmission electron microscopy analyses revealed that PROH provided the most efficient preservation of the ovarian tissue ultrastructure and thus, is suggested for use in agouti ( Wanderley *et al*., 2012 ).

Another study performed a cryopreservation protocol based on solid surface vitrification of *D. leporina* ovarian tissue. It was verified that regardless of the CPA used (DMSO 3.0 M or 6.0 M, EG 3.0 M or 6.0 M, and a combination of both agents), it was possible to preserve up to 70% of the follicular morphology. Moreover, DNA fragmentation was not observed in any of the groups exhibiting preserved follicular viability similar to that observed in the non-vitrified group ( Praxedes, 2017 ).

In addition, the first ovarian tissue xenograft of non-vitrified and vitrified fragments of *D. leporina* was reported in 2017. Grafts are tools that can be used to measure the survival of tissue after cryopreservation and, in case of ovarian grafts, to obtain knowledge regarding follicular dynamics of various species. Praxedes *et al.* (2018) using a combination of DMSO and EG in immunosuppressed mice for both the non-vitrified (80%) and vitrified (16%) groups observed that ovarian activity was recovered after 20.6 ± 8.6 days of xenografting. The recovery of ovarian activity was characterized by the presence of typical signs of proestrus and estrus, associated with the increase in E_2_ concentrations in recipient severe combined immunodeficiency (SCID) mice. Microscopically, primordial, primary, transitional, and secondary follicles, corpora lutea, and hemorrhagic body were observed in the grafts exhibiting normal morphology for the species studied (Praxedes *et al*., 2017).

Even with the advances in studies on reproductive biotechniques ( Tab. 4 ) aimed at conserving the genetic material of female agouti, it is necessary to improve the existing protocols that allow better rates of preservation of morphological integrity and viability of follicles before they become atresic. These studies are needed not only to safeguard genetic material, but also for use in other biotechniques, such as *in vitro* fertilization and cloning.

**Table 4:** State of the art of assisted reproductive techniques **(**ARTs) applied for agoutis’ (*Dasyprocta spp.)* females.

Species	ARTs	Source
*Dasyprocta leporina*	Monitoring the estrous cycle	Campos *et al*., (2015) Singh *et al.,* (2016)
	Gestational monitoring	Sousa *et al.,* (2017) Oliveira *et al*., (2017)
	Induction of estrus	Peixoto (2016)
	Cryopreservation of ovarian tissue	Wanderley *et al.,* (2012) Praxedes (2017)
	Xenograft of fresh and vitrified ovarian tissue	Praxedes et al., (2017)
*Dasyprocta prymnolopha*	Monitoring the estrous cycle	Guimarães *et al.,* (1997)
	Gestational monitoring	Souza *et al*., (2012)

However, in wild animals, cryopreservation of female gametes and conditions necessary for the complete development of *in vitro* PFs are still not well established. Thus, many studies are aimed at developing and adapting efficient *in vitro* culture systems to evaluate the effect of different substances (gonadotropins and intra-ovarian factors) on initial oocyte development for obtaining information about the mechanisms involved in folliculogenesis.

### Conservation and culture of agouti somatic tissue

The establishment of somatic cryobanks has been suggested as an important tool for conservation of endangered ( Loi *et al*., 2001 or zootechnically valuable species ( Loi *et al*., 2001 , Pereira *et al*., 2013 ), as an alternative to the conservation of animal biodiversity. It allows the optimization of other reproductive biotechniques in association with nuclear somatic cell transfer (SCNT, also known as cloning). In agouti, studies focused on the formation of biobanks derived from somatic tissue are still nascent and little is known about the use of this genetic source.

Thus, aimed at the formation of germplasm banks, a study used peripheral ear tissue of *D. leporina* and analyzed different techniques of vitrification (solid surface and conventional vitrification) for the conservation of somatic samples. Vitrification consisted of exposing the fragments in DMEM medium supplemented with 20% DMSO, 20% EG, 0.25 M sucrose, and 10% fetal bovine serum for 5 min. Based on histological analyses, it was observed that solid surface vitrification better preserved the somatic tissue ( Costa *et al*., 2015 ).

Thus, the interest in various tissue sources is primarily based on the possibility of using different cell types as a nucleus donor cell in SCNT ( Arat *et al*., 2011 ). Therefore, samples derived from the skin are widely used for tissue preservation in addition to the formation of cryobanks, reproduction by SCNT, and pluripotency studies ( Borges *et al*., 2015 ).

## Final Considerations

The *Dasyprocta spp.*, besides being considered an alternative source of protein, has great potential as an experimental model for studying reproductive biotechniques and the formation of germplasm banks. However, it presents distinct limitations. The evolution and adaptation of different protocols to the characteristics of each species is not characterized. Moreover, it should be noted that very little is known about the morphophysiological characteristics of male and female agouti. This knowledge is essential for future application in sustainable production systems as well as for the development and improvement of protocols that guarantee the maintenance of cellular viability and allow the conservation, multiplication, and preservation of the biodiversity of these species and other related species.
